# A Comparison of Various Algorithms for Classification of Food Scents Measured with an Ion Mobility Spectrometry

**DOI:** 10.3390/s21020361

**Published:** 2021-01-07

**Authors:** Georgy Minaev, Philipp Müller, Katri Salminen, Jussi Rantala, Veikko Surakka, Ari Visa

**Affiliations:** 1 Faculty of Information Technology and Communication Sciences, Tampere University, 33720 Tampere, Finland; philipp.muller@tuni.fi (P.M.); jussi.rantala@tuni.fi (J.R.); veikko.surakka@tuni.fi (V.S.); ari.visa@tuni.fi (A.V.); 2 R&D and Innovation Services, Tampere University of Applied Sciences, 33230 Tampere, Finland; katri.salminen@tuni.fi

**Keywords:** scent classification, nearest neighbour, electronic nose, ion mobility spectrometry

## Abstract

The present aim was to compare the accuracy of several algorithms in classifying data collected from food scent samples. Measurements using an electronic nose (eNose) can be used for classification of different scents. An eNose was used to measure scent samples from seven food scent sources, both from an open plate and a sealed jar. The *k*-Nearest Neighbour (*k*-NN) classifier provides reasonable accuracy under certain conditions and uses traditionally the Euclidean distance for measuring the similarity of samples. Therefore, it was used as a baseline distance metric for the *k*-NN in this paper. Its classification accuracy was compared with the accuracies of the *k*-NN with 66 alternative distance metrics. In addition, 18 other classifiers were tested with raw eNose data. For each classifier various parameter settings were tried and compared. Overall, 304 different classifier variations were tested, which differed from each other in at least one parameter value. The results showed that Quadratic Discriminant Analysis, MLPClassifier, C-Support Vector Classification (SVC), and several different single hidden layer Neural Networks yielded lower misclassification rates applied to the raw data than *k*-NN with Euclidean distance. Both MLP Classifiers and SVC yielded misclassification rates of less than 3% when applied to raw data. Furthermore, when applied both to the raw data and the data preprocessed by principal component analysis that explained at least 95% or 99% of the total variance in the raw data, Quadratic Discriminant Analysis outperformed the other classifiers. The findings of this study can be used for further algorithm development. They can also be used, for example, to improve the estimation of storage times of fruit.

## 1. Introduction

Artificial sensing of scents can be done with various methods such as gas chromatography mass spectrometer (GC-MS) or chemical sensors. All the solutions share a basic functional principle of detecting volatile organic compounds (VOCs) and using databases and algorithms to classify them. One of the most prominent technologies are electronic noses (eNoses) that can classify and detect various gases with high accuracy. They mimic both the biological sense of smell and human brain processing the sensory information [1]. An eNose uses a sensor array, a signal processing unit, a reference database, and pattern recognition software [2].

VOC analysis has a long tradition in several industrial applications, including food industry, food safety [3,4], perfumery, cosmetics, agriculture [5], and medicine [2,6]. Previous work in analysing data measured by eNoses has concentrated on supervised learning methods. Supervised learning methods use measurements from known samples (so-called training samples) to learn a mapping function, which allows the classification of unlabelled samples (so-called test samples). An overview of classification algorithms based on supervised learning methods can be found in [1]. Methods used in the previous research include principal component analysis (PCA; e.g., in [7]), linear discriminant analysis [8], canonical discriminant analysis [9], discriminant functions analysis [10], hierarchical cluster analysis [11], cluster analysis [12], *k* Nearest Neighbour [6], support vector machine [13], fuzzy artificial neural networks [14], and multilayer perceptron (MLP)-type classifiers [15].

The eNose used in the current work is based on ion mobility spectrometry (IMS) [6]. Most IMS sensors do not age significantly, as they rely on radioactive sources, with half-lives of several hundred years, for ionizing the molecules. This means that the IMS sensors experience signal drift mainly due to environmental changes. This is a clear advantage in comparison to other eNoses (e.g., metal-oxide sensors). The ChemPro 100i uses an Americium-241 source for ionization, which is similar to the radioactive sources used in ionization-type smoke detectors. In addition, ChemPro 100i is light-weight, has low-power consumption enabling on-site measurements, it is fast, has high accuracy for selected substances (e.g., nitro-organic explosives), low price, and an easy user interface. One significant advantage of IMS is that air can be employed as a carrier gas, which supports its applicability in real-world measurements [16,17,18]. IMS technologies are especially suitable for environmental analysis outside laboratory. For example, recently, IMS data was successfully used for indoor localization [19,20].

Despite the previous work in the area, the data collected by IMS devices is still subject to challenging variations, difficulties of interpretation, and complexity. Recent reviews indicate significant need to develop analytical methods, especially for data sets measured outside laboratories [16,17,18]. Identified issues in data collected outside laboratories include higher detection limits, lower sensitivity, sample contamination, and the strong impact of environmental factors (e.g., other target substances and moisture) on the signal. Because of this, commercial applications of IMS in environmental analysis are targeted to detect only one compound or compound group (e.g., explosives) [21].

Furthermore, the analysis of the measurement signal still requires development, especially for situations where the measurement is conducted in real life-like conditions with little, if any, attempts to control the environmental noise. Both selectivity and sensitivity are identified as significant problems in environmental analysis using IMS [16]. Research and development of classification algorithms is needed to address these challenges.

The volatile organic and aromatic compounds emitted by food aromas are complex. For high accuracy [22], they are often measured with gas chromatography (GC), which requires complicated pre-treatments before the analysis creating long detection times. Combining GC with IMS partly overcomes these challenges, but still ends up having a single detection time varying from 2 min up to 30 min [23] and retention time between 100 and 900 s [24]. Food analysis conducted with rapid and portable IMS has significant [3,4,25] applications potential, including analysis of food composition, process control, authentication, food safety (including chemical safety and residuals), estimation of freshness, and flavour profile. However, the problems related to the use of IMS measurements outside laboratory still hinder the widespread adaptation of the technology.

This paper addresses these issues by comparing 19 different classifiers with different parameter settings resulting in 304 variations. The classifiers were applied to two data sets collected from food samples. One collected from headspace, where the environmental conditions were controlled, and the other collected from an office desk without explicit attempts to control the environmental conditions (see, for example, [26,27,28] for detailed description of the data collection). The objective of the paper was to present and compare several analytical tools for improving the classification accuracy and speed for food scents also in the presence of environmental noise. The results could facilitate food quality analysis in different environmental conditions.

The key question was how to improve matching between labelled and unlabelled samples. Often, in previous studies distance-based learning algorithms, such as the *k*-Nearest Neighbour (*k*-NN), are studied for classifying scent data [6,29,30]. In general, it is important to proceed with as few labelled training samples as possible. Previous studies, generally, used only a few distance measures or simply relied on the Euclidean distance, but the performance of *k*-NN can be significantly improved by using alternative distance measures [19,31]. In the current paper an extensive list of 67 different distance measures were tested within the *k*-NN classifier. In addition, this present work studied several alternative classifiers and compared them to the *k*-NN algorithm. In [6] the possibility of using *k*-*d* tree search (please note that the *k* in *k*-*d* tree search refers to the dimension of the measurements rather than the number of nearest neighbours, for details see [32]) instead of exhaustive search for finding the *k* nearest neighbours was studied. The *k*-*d* tree search showed similar misclassification rates, but required only ≈15% of the evaluation time when tested with the two datasets [6]. Furthermore, [6] found no significant influence of the number of nearest neighbours on the misclassification rate. Therefore, for simplicity, only NN (i.e., k=1) with exhaustive search is studied in this paper.

The misclassification rates of *k*-NN with different distance measures were then compared with the misclassification rates of 19 different alternative classifiers. For all classifiers different parameters settings were used. In total 304 different combinations of classifiers with different parameters were applied to the raw IMS data. For the PCA transformed data, explaining 95% and 99% of the total variance in the data, 292 classifiers with different parameters were tested. The slightly smaller number of tested classifiers was due to the smaller number of features for training and test vectors. This paper presents only the best classifiers and distance measures as compared with the Euclidean distance. Results from all tested classifiers can be found in the Supplementary Material. The selected list of classifiers includes Quadratic Discriminant Analysis, MLPClassifier, and C-Support Vector Classification (CSV).

The present work is organized as follows. The IMS fingerprint-based classification with Nearest Neighbour classification, Quadratic Discriminant Analysis, Artificial Neural Network, C-Support Vector Classification, Principal Component Analyses, and Cross Validation are explained in Section 2. The ChemPro 100i eNose, which was used for data collection, and the data are described in Section 3. All experiments, tests and results are shown and described in Section 4. Section 5 concludes the paper and gives an outlook.

## 2. IMS Fingerprint-Based Classification

### 2.1. Nearest Neighbour Classification

The Nearest Neighbour (NN) algorithm is a popular and simple approach for classification. NN classification shows similar evaluation time and similar or lower misclassification rates as the *k*-NN variations studied in [6], depending on the used data set. The reason for choosing the NN was the limited number of available training samples.

The NN classifier compares the test vector with all training vectors in order to find the most similar one. The scent label of the most similar training vector is then used as a label for the test vector.

The Euclidean distance is traditionally used for measuring the similarity of the test and training samples (e.g., in [6]), but various alternative distance measures exist that can be used in the NN classification. In this paper, we present the results of four best distance measures out of 66. The four distance measures and the link to the full list of measures are described in Section 4.2.

### 2.2. Quadratic Discriminant Analysis

Quadratic Discriminant Analysis (QDA) is a well-known fundamental supervised classification method in statistical and probabilistic learning [33]. QDA works with likelihoods and priors but deals with maximizing the posterior of classes. The logarithm of the posterior is defined as

(1) $$\log P\left(y=l|x\right)=\frac{1}{2}{\left(x-\mu_{l}\right)}^{t}\sum_{l}^{-1}\left(x-\mu_{l}\right)+\log P\left(y=l\right)$$

where *l* is a class, $x\in R^{d}$, *d* is data vector dimension, *x* is sample vector, $\mu_{l}$ is mean of class *l*, and ${\left(x-\mu_{l}\right)}^{t}\sum_{l}^{-1}\left(x-\mu_{l}\right)$ corresponds to Mahalanobis distance. The Mahalanobis distance shows how close the test vector *x* is to the cluster mean $\mu_{l}$. Notation is taken from https://scikit-learn.org/stable/modules/lda_qda.html#id1.

### 2.3. Artificial Neural Network

**Figure 1:**
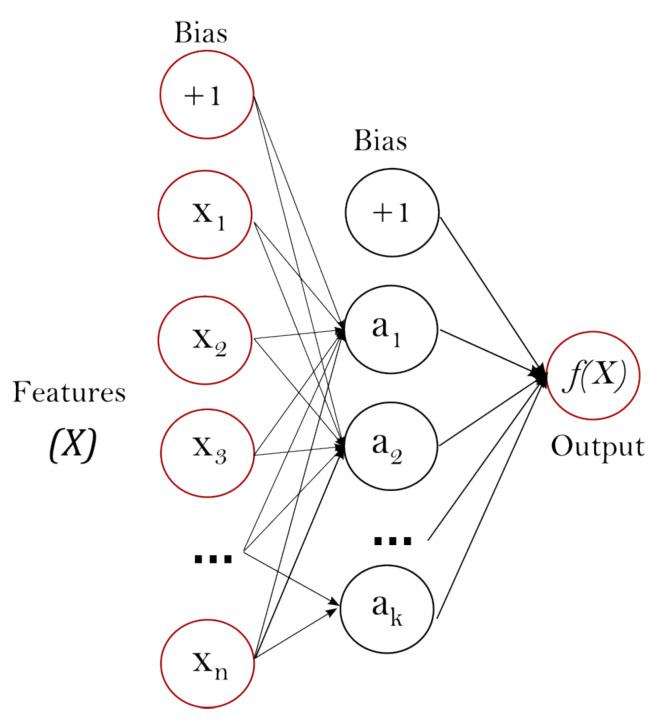
Example of single hidden layer neural network. Layer *X* is input layer, layer *a* is hidden layer. The image is taken from https://scikit-learn.org/stable/modules/neural_networks_supervised.html.

Artificial Neural Network (ANN) or “neural networks” is motivated by the fact that human brain computes differently from conventional digital computer [34]. The brain is a highly complex, non-linear and parallel computer. Neurons are part of the human brain. An ANN is designed to imitate the way the human brain works. Therefore, it consists of artificial neurons. Figure 1 shows the structure of a single layered neural network.

The main highlight points of the basic features of a neural network are:

1. Each neuron has an activation function. The activation function defines the output of a neuron.
2. A network has one or more layers, which are hidden from both input and output layers.
3. There is a high level of connectivity between neurons.

Several ways for training neural networks exist. One of them is ‘lbfgs’, which is an optimizer in the family of quasi-Newton methods. In this paper, only the ’lbfgs’ method was used to train a single layer neural network. An explanation of quasi-Newton methods can be found in [34,35].

### 2.4. C-Support Vector Classification

C-Support Vector Classification (C-SVC) takes vectors in two classes $x_{i}\in R^{n},i=1,\ldots ,l$ and a characteristic vector $y\in R^{l}$, such that $y_{i}\in 1,-1$ [36]. The goal of C-SVC is to solve the optimisation problem

(2) $$\begin{array}{c} \min_{\omega ,b,\xi }\frac{1}{2}\omega^{T}\omega +C\sum_{i=1}^{l}\xi_{i}\\ subject\;to\;y_{i}\left(\omega^{T}\phi \left(x_{i}\right)+b\right)\geq 1+\xi i_{i}\\ \xi_{i}\geq 0,i=1,\ldots ,l\\ \omega =\sum_{i=1}^{l}y_{i}\alpha_{i}\phi \left(x_{i}\right) \end{array}$$

where C>0 is a regulation parameter, $\phi (x_{i})$ maps $x_{i}$ into a higher-dimensional space. The strength of regularization is inversely proportional to *C*.

Finally, the decision function is

(3) $$sgn\left(\omega^{T}\phi \left(x\right)+b\right)=sgn\left(\sum_{i=1}^{l}y_{i}\alpha_{i}K\left(x_{i},x\right)+b\right)$$

### 2.5. Principal Component Analyses

Principal component analysis (PCA) is used to project data onto a smaller number of dimensions, improve the calculation speed and remove potential correlation between features. The original features are projected onto *d* linearly uncorrelated variables [37] (p. 580). The PCA method with the training data $X=\{x_{1},\ldots ,x_{N}\}$ and *d* features works as follow [37] (p. 568), [6,19]:

1. Calculate *d*-dimensional mean vector μ and *d*-by-*d* dimensions covariance matrix C for training data set X.
2. Eigenvectors and eigenvalues of C are calculated and sorted according to decreasing eigenvalues.
3. A subset of these eigenvalues is chosen, for instance, the first *m* eigenvalues form *d*-by-*m* matrix A (*m* eigenvectors as columns of A).
4. PCA-transformed data $Y=\{y_{1},\ldots ,y_{N}\}$ is defined as $y_{i}=A^{T}\left(x_{i}-\mu \right)$, where each $y_{i}$ has *m* variables.

A test sample is transformed into the same format as the training data using $y_{i}=A^{T}\left(x_{i}-\mu \right)$.

The choice of the *m* principal components affects how much of the total variance in X is explained by the transformed data Y. In theory all principal components can be used (i.e., m=d) to explain the total variance in X. However, normally small subsets (m<d) are chosen in an attempt to remove noise from the data. Here small subsets are chosen that explain at least 95% and 99% of the total variance of the training data.

In this paper NN, ANN, C-SVC and other classifiers, which did not show good enough results to be shown here, were applied to PCA-transformed data.

### 2.6. Cross Validation

Cross-validation is used to get access to the generalization ability of predictive models and prevent overfitting [34,38]. Overfitting yields a model which fits the training data very well but is unable to classify unseen data correctly. The generalization ability of a model is a way of describing the possibility of the model to classify unseen data. Cross validation has been developed to check the ability of a model for generalization. In the present paper a setup inspired by *m*-fold cross validation was used. Details can be found in Section 3.

## 3. Data

In the present paper, the same dataset as in [6] was used. The dataset is freely available [26]. It consists of IMS readings of seven food scent sources: cinnamon, coffee, grape, lemon peel, pineapple, strawberry, and vanilla. The readings were collected with a ChemPro 100i [39] eNose from Environics Ltd (Mikkeli, Finland). The ChemPro 100i is an aspiration-type Ion Mobility Spectrometer (aIMS) that ionises incoming air and pushes the ionized air through an electric field in its IMCell equipped with IMS sensors.

Each source was presented to the eNose in two ways, on a plate and in a sealed jar. For the presentation from plate, the scent source was placed approximately 2–3 cm from the eNose inlet. The scented air was sucked in by the eNose using a flow generated by a rotary vane pump. After ionization, ions were measured by seven separate electrode pairs. Depending on their charge and their flight times, different ions were measured as currents by different electrodes. This means that any electrode measured ions with the same charge and flight times within in a certain interval. The seven positive and seven negative currents measured by the electrode pairs were interpreted as a scent source’s 14-dimensional “fingerprint” and used as measurement data. This means that each measurement was represented by $x=\{x_{1},\ldots ,x_{14}\}$ and a corresponding scent label y.

For the presentation from a sealed jar, a custom cap with inlet and outlet was attached to the jar for controlling air flow through the odorous headspace. This setup was chosen to minimize the impact of environmental noise on the IMS fingerprints (VOCs measured from the sealed jar were pure while scents measured from plate were “contaminated” by molecules in the surrounding air). It is noted that our analysis showed that the presentation method had a significant impact on the IMS readings.

**Figure 2:**
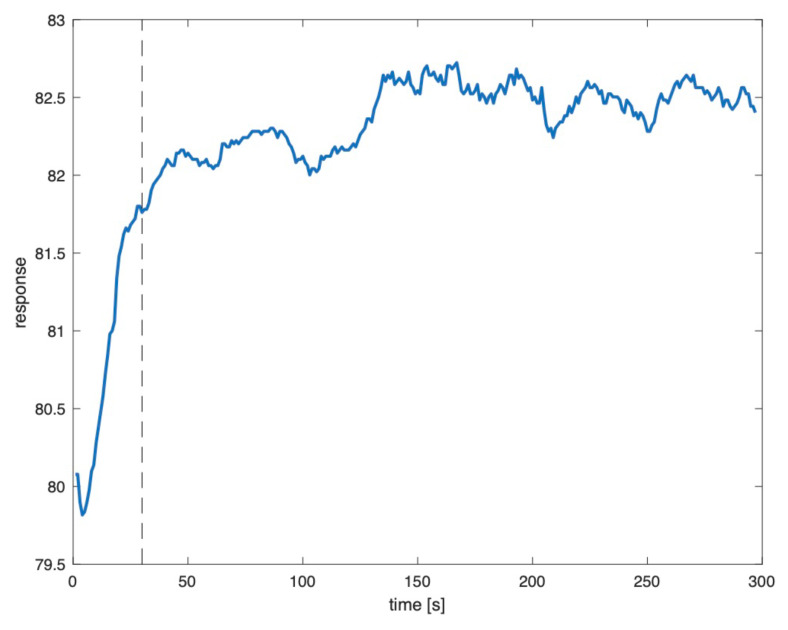
Example of transition phase (left of dashed line) and stable phase (right of dashed line) on IMS channel 1 for cinnamon measured from jar [6].

The dataset consisted of five measurement sets for each food scent source and the two presentation methods, meaning that there were 70 measurement sets. The scent source volume was around 5 mL in all tests. Each set consisted of 5 min IMS fingerprints recorded with a sampling rate of 1 Hz. The reason for collecting time series data instead of snap shots was that IMS readings showed temporal variation and both transient and stable phase. The transient phase is the phase in which the measured currents are unstable with either an upward or a downward trend. Its length depended on the scent, the presentation method, and even varied between channels (i.e., electrodes) of the same scent. Typically, transition lengths of approximately 30 s were observed [6]. Figure 2 shows an example of an IMS channel reading with the transient phase ending after approximately 35 s (dashed vertical line). Between collecting two measurement sets a break of 3 min was kept in order for the IMS reading to return to the baseline and reproduce the transient phase in all measurement sets.

The use of five measurement sets per scent source and presentation method enabled the use of separate training and test sets. A setup inspired by 5-fold cross validation was used. In the first run all measurement sets with identifier (ID) 1 formed the test set and all measurement sets with IDs 2 to 5 formed the training data. In the second run all measurement sets with ID 2 were used as test data and all sets with IDs 1 and 3 to 5 were used as training data. In runs 3 to 5 similar setups were used. The misclassification rates, fitting times and prediction times (see Section 4) were computed by averaging over the misclassification rates and times of the five runs.

## 4. Experiments

### 4.1. Nearest Neighbour Using Euclidean Distance

**Figure 3:**
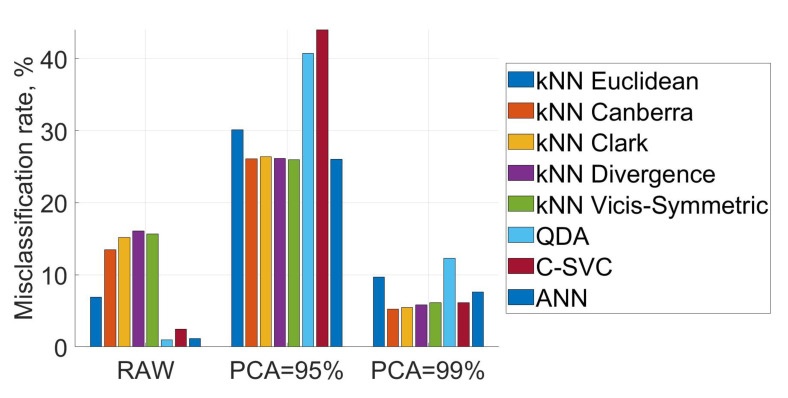
Misclassification rates of eight different classifiers. RAW indicates that raw IMS data was used and PCA indicates that PCA-transformed data explaining x% of the total variance in raw IMS data was used for classification. The presented misclassification rates are average rates over the five runs.

The first aim of this paper was to study the impact of preprocessing IMS fingerprints on the misclassification rates with the Euclidean distance as the distance measure for the NN classifier. Figure 3 shows misclassification rates for using raw IMS data (raw = no preprocessing techniques applied [20]), and principal components that explained at least 95% and 99% of the total variance in the IMS data. Figure 3 and Table 1 show the average results over the five runs. Using raw IMS data yielded the lowest average misclassification rate followed by PCA-transformed data explaining at least 99% of the total variation. Using only three principal components (95%) yielded a significantly higher misclassification rate, and cannot be recommended.

**Figure 4:**
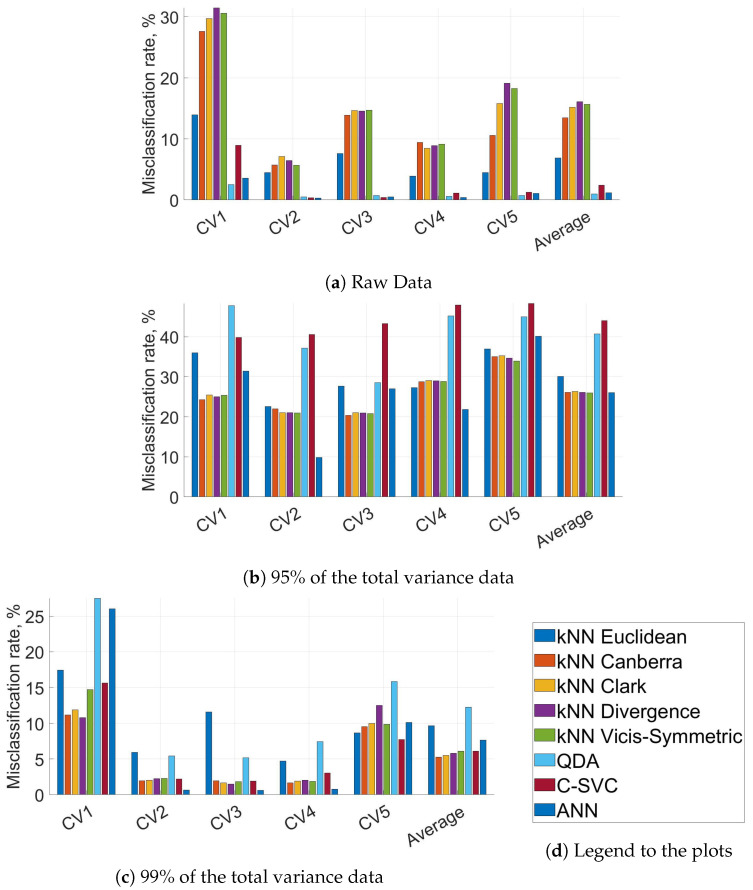
Misclassification rates of all runs (CV1 to CV5) and average misclassification rates. Legends are the same as in Figure 3.

Figure 3 and Table 1 show also the misclassification rates of *k*-NN with a selection of different distance measures and different classifiers. The best shown methods with raw data are QDA, MLP classifier with hidden layer size 8 and C-SVC with C=0.025. Canberra and Clark applied to PCA-transformed data explaining 99% of the variance yielded better accuracy while for Euclidean it decreased accuracy compared to raw data. A detailed presentation of the misclassification rates in different runs can be found in Figure 4. It shows that in the first run misclassification rates are significantly higher than in the other four runs (for raw data and PCA-transformed data explaining 99% of the total variance). This is in line with the findings of [6] and can be attributed to the fact that the IMS channel readings did not return to baseline levels for all analysed food scent sources. A more detailed explanation can be found in [6]. For PCA-transformed data explaining 95% of the total variation the misclassification rates are high for every classifier, in every run. Thus, using this data for classification is not recommended.

### 4.2. Nearest Neighbour Using Alternative Distance Measures

The aim of this section was to find the best distance measure for the NN classifier with respect to the misclassification rate. In total 67 distance measures were studied, including Euclidean distance as a reference measure. The full list of distance measures can be found in [40]. The best four distance measures were chosen based on the experiment with at least 99% of the total variance explained by the data. The Euclidean distance and the four alternative distance measures are summarized in Table 2 and their average misclassification rates are shown in Table 1. The misclassification rates of the remaining 62 distance measures can be found in the Supplementary Material.

### 4.3. Artificial Neural Network

**Figure 5:**
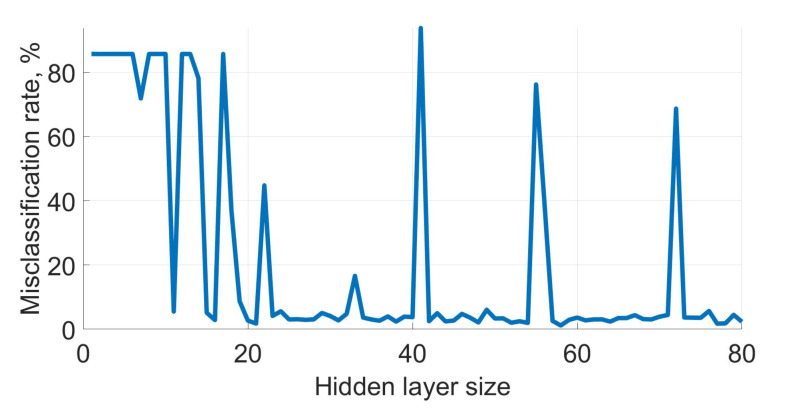
Misclassification rates of single layer ANN with varying number of nodes.

The goal of this section was to study the behaviour of artificial neural networks for scent classification and find the hidden layer dimension yielding the lowest misclassification rate. The number of hidden layers was varied from a single layer to seven hidden layers with ten nodes each. The single hidden layer was tested with 1 to 80 nodes, and the average misclassification rates of the single hidden layer ANN are shown in Figure 5.

Figure 5 shows that no significant improvements could be achieved by increasing the number of nodes above 20 in the single layer network. There were outliers when the ANN included 22, 41, 55, 66 and 72 nodes. Table 3 shows that these high misclassification rates correspond to short relative fit time in comparison with neighbouring values. This implies that at 22, 41, 55, 66 and 72 local minima were found.

Table 1 shows that the lowest misclassification rate with PCA 99% transformed data is shown by MLPClassifier with hidden single layer size of 63.

Tests with multi hidden layers perceptron (MPL) classifiers performed worse and are, therefore, omitted from this paper. The default activation function was ‘relu’, which returned f(s)=max(0,x). The solver for weight optimization is from the family of quasi-Newton methods ‘lbfgs’. The following MLP classifiers were tested:

- MLP with single layer ranging from (1) to (80)
- MLP with two layers ranging from (1,1) to (10,5).
- MLP with three layers was tested with layers (1,1,1), (2,2,2), (3,3,3), (4,4,4), (5,5,5), (6,6,6), (7,7,7), (8,8,8), (9,9,9) and (10,10,10)
- MLP with four layers was tested with layers (1,1,1,1), (2,2,2,2), (3,3,3,3), (4,4,4,4), (5,5,5,5), (6,6,6,6), (7,7,7,7), (8,8,8,8), (9,9,9,9) and (10,10,10,10)
- MLP with five layers was tested with layers (1,1,1,1,1), (2,2,2,2,2), (3,3,3,3,3), (4,4,4,4,4), (5,5,5,5,5), (6,6,6,6,6), (7,7,7,7,7), (8,8,8,8,8), (9,9,9,9,9) and (10,10,10,10,10)
- MLP with six layers had layer sizes (10,10,10,10,10,10)
- MLP with seven layers had layer sizes (10,10,10,10,10,10,10)

The results can be found in the Supplementary Material.

### 4.4. C-Support Vector Classification

C-Support Vector Classification performed well with raw IMS data, achieving an average misclassification rate of 2.434%. This was lower than the best result showed with PCA-transformed data explaining 99% of the total variance in the data. It even outperformed the ANN with a single layer of size 63 (3.664% average misclassification rate).

### 4.5. Quadratic Discriminant Analysis

Quadratic Discriminant Analysis (QDA) yielded the best overall result with a misclassification rate of 1.004% when applied to raw data. QDA performed significantly worse with PCA transformed data (see Table 1) and significantly worse than other classifiers applied to PCA-transformed data.

### 4.6. Other Classifiers

A total of 19 different classifiers were studied. Classifiers that were tested included

- Gradient Boosting Classifier with 100 estimators, a learning rate of 1.0, and a maximal depth from 1 to 10
- Random Forest Classifier with a maximal depth ranging from 1 to 190, number of estimators from 10 to 190, and maximal features from 1 to 14
- Decision Tree Classifier with maximal depths ranging from 1 to 10
- SGD Classifier
- SVC with max iterations set to −1, kernels “linear” and “rbf”, C = 0.025, gamma = 2, C = 1
- Perceptron
- Passive Aggressive Classifier
- Ada Boost Classifier
- Quadratic Discriminant Analysis
- Gaussian NB
- NearestCentroid
- Bernoulli NB with α = 0.1
- Lasso with maximal iterations set to 1,000,000, α = 0.1
- LassoLars with α = 0.1 and maximal iterations set to 1,000,000
- Orthogonal Matching Pursuit
- Orthogonal Matching Pursuit CV
- PLS Regression with Number of components to keep 1, 2 and 3

However, they all performed worse than the classifiers presented in Table 1. Therefore, these classifiers were not discussed in detail. Interested readers can find the results of all tested classifiers and parameter settings in the Supplementary Material.

## 5. Conclusions and Outlook

The goal of this paper was to provide an extensive comparison of machine learning techniques used for accurate and rapid classification of volatile organic compounds (VOCs) emitted by food samples and measured by IMS. The following conclusions present best classifiers and distance measures in terms of misclassification rate and time. The lowest misclassification rates (see Table 1) for raw data were achieved by using Quadratic Discriminant Analysis and MLP classifier with hidden layer including 58 neurons. The lowest misclassification rate with PCA-transformed data explaining 99% of the total variance in the IMS data was achieved by using MLP classifier with a single hidden layer size of 63 neurons. The *k*-NN classifier with k=1 and either Clark, Canberra, Divergence or Vicis-Symmentric $\chi^{2}$ distance as similarity measure yielded lower misclassification rates when applied to PCA-transformed data explaining 99% of the total variance than when applied to raw data. This is in contrast to the results for the *k*-NN using Euclidean distance, QDA, and SVC. One of the reasons for that behaviour is that Canberra, Clark, and Divergence distances normalize in each feature. Normalization of Canberra and Clark is done by $|P_{i}\left|+\right|Q_{i}|$, while Divergence normalization is done by ${\left(P_{i}+Q_{i}\right)}^{2}$. Overall, the results clearly show that the misclassification rate remains low even without using 99% PCA-transformation, and that 95% PCA-transformation results in the highest misclassification rates.

Prediction time for the tested Nearest Neighbor methods directly depended on the size of the training set because the exhaustive search was used. A simple yet effective way to significantly reduce the prediction time is to replace the exhaustive search by *k*-*d* tree search. In [6] it was shown that the prediction time of *k*-*d* tree search was only approximately 15% of the prediction time of the exhaustive search. At the same time, it had no significant impact on the classification accuracy. Since Clark, Canberra, Divergence, and Vicis-Symmentric $\chi^{2}$ distances significantly increase the prediction time compared to the Euclidean distance, switching to *k*-*d* tree search might be necessary.

The IMS fingerprints used for classification in this paper are vectors of fixed dimension 14. In applications, where environmental noise creates significant drift to the signal, it may be necessary to use other sensory data from IMS to keep the misclassification rate low and ensure rapid classification. If further sensor measurements, such as temperature or humidity, would be added, then the vector dimension would increase only slightly. Therefore, considering alternative methods for high-dimensional data is unnecessary. Increasing the size of training data affects the fitting but not the evaluation time of an MLP classifier. Fitting MLP classifiers is very time consuming. This is unproblematic only if the fitting can be done offline and needs to be done only once, meaning that no new data is added to the training set at some later stage. At the same time, predictions can be obtained significantly faster with the MLP classifier than with the Nearest Neighbour method. Thus, the choice between these classifiers depends on various parameters, such as size of training data, computational resources for model fitting and predictions, and application (i.e., online or offline).

In summary, the research on food analysis outside laboratory faces significant problems related to the variation in hardware (e.g., computers, IMS) and data quality (e.g., the amount of environmental drift), which both significantly affect the selectivity and sensitivity of the measurement [16]. Furthermore, most previous studies have used only two or three different classifiers providing an insufficient understanding of the analytical methods required especially for measurements outside the laboratory. The current paper provides a framework of classifiers that can be used in food analysis applications, such as quality measurements (e.g., grains, eggs, meat, fish, and seafood as well as compounds in dairy products), food authentication etc. [2,43,44,45].

To our knowledge, there is no earlier research in comparing the functionality of classification algorithms with this extent. Considering the low misclassification rates of Quadratic Discriminant Analysis, Artificial Neural Networks, and C-Support Vector Classification, it seems that machine learning methods enable reliable classification based on IMS measurements. Because the current measurements were carried out in a laboratory environment, further work is needed in more natural environments for which our findings pave way. For future research, this hypothesis could be tested with even larger datasets.

With such datasets various combinations of promising classifiers will be tested. By using voting schemes, in general, more robust results can be achieved. In the literature this is often referred to as boosting. For example, Quadratic Discriminant Analysis, MLP classifier with 63 neurons in the hidden layer, and C-Support Vector Classification with C = 0.025 could be combined in a voting scheme. If all three or two of the classifiers yield the same label for a VOC test sample, then this label will be used for the sample. However, if all three classifiers yield different labels, then the best option might be do inform the user that the test sample cannot be classified unambiguously. Besides the three best classifiers, other, weaker classifiers, could be combined (e.g., nearest neighbor classifiers with different distance metrics) into one classifier and compared. At the same time, it could be checked how to handle test data for which no training data is available. It is crucial to avoid falsely classifying such data as one of the foods from the training data.

## Figures and Tables

**Table 1:** Times for fitting mapping functions (Raw Fit time) and predicting times of test data (Raw Predict time) are relative to times for NN classifier with Euclidean distance. All values were averaged over the five runs. Misclassification rates are given for classification based on raw IMS data and based on PCA-transformed data explaining 95% and 99% of the total variance in the raw data. Large relative times for Canberra, Clark, Divergence, Vicis-Symmetric $\chi^{2}$ are due to their implementations. Results for other distance measures and classifiers can be found in the Supplementary Material.

ID	Name	Raw Fit Time	Raw Predict Time	Raw	PCA 95%	PCA 99%
1.	Euclidean	1	1	6.894	30.114	9.674
30.	Canberra	18.885	189.334	13.456	26.106	5.258
38.	Clark	24.838	293.959	15.168	26.368	5.508
54.	Divergence	21.615	127.758	16.102	26.136	5.816
60.	Vicis-Symmetric $\chi^{2}$	20.841	130.258	15.676	25.994	6.122
	Quadratic Discriminant Analysis	0.103	0.014	1.004	40.736	12.290
	SVC C=0.025	4.894	0.561	2.434	44.010	6.114
	MLPClassifier hidden layer size 58	1719.458	0.045	1.168	26.056	7.640
	MLPClassifier hidden layer size 63	1881.380	0.047	3.082	22.494	3.664

**Table 2:** Selected distance measures. d(P,Q) is the distance between vectors *P* and *Q*.

ID	Name	d(P,Q)	Source
1.	Euclidean $\begin{array}{l} p=2\\ r=2 \end{array}$	${\left(\sum {\left|P_{i}-Q_{i}\right|}^{p}\right)}^{1/r}$	[41] chapter 17.2
30.	Canberra	$\sum \frac{\left|P_{i}-Q_{i}\right|}{\left|P_{i}\right|+\left|Q_{i}\right|}$	[41] chapter 17.1
38.	Clark	${\left(\frac{1}{N}\sum {\left(\frac{\left(P_{i}-Q_{i}\right)}{\left(\left|P_{i}\right|+\left|Q_{i}\right|\right)}\right)}^{2}\right)}^{1/2}$	[41] chapter 17.1
54.	Divergence	$2\sum \frac{{\left(P_{i}-Q_{i}\right)}^{2}}{{\left(P_{i}+Q_{i}\right)}^{2}}$	[42]
60.	Vicis-Symmetric $\chi^{2}$ 1	$\sum \frac{{\left(P_{i}-Q_{i}\right)}^{2}}{\min{\left(P_{i},Q_{i}\right)}^{2}}$	[42]

**Table 3:** Errors of fruit’s misclassification rates of single hidden layer ANN. The misclassification rate values are average values of 5 experiments. The columns are hidden layer network size, relative time to fit, relative time to predict and misclassification rate in percent. Relative time is the time in comparison with NN with Euclidean distance.

Size	Fit Time	Predict Time	Misclassification Rate
40	1638.190	0.031	3.770
41	4.667	0.030	93.814
42	1857.807	0.033	2.504
54	1964.511	0.038	1.980
55	903.012	0.041	76.206
56	1063.717	0.043	40.030
57	1468.125	0.041	2.604
71	1664.452	0.051	4.436
72	477.978	0.052	68.704
73	2611.069	0.055	3.656

## Data Availability

Data is available at https://etsin.fairdata.fi/dataset/5e5eecec-fd69-40ab-ad44-216ceac474df or by contacting Philipp Müller. License: Creative Commons Attribution 4.0 International (CC BY 4.0).

## Supplementary Materials

The following are available online at https://www.mdpi.com/1424-8220/21/2/361/s1, A document containing the miclassification rates, run and fix times of all tested classifiers that were not presented in this manuscript.
