# Supplementary figures and images for: A Comparison of Various Algorithms for Classification of Food Scents Measured with an Ion Mobility Spectrometry
Source: Sensors (Basel). 2021 Jan 7;21(2):361. doi: 10.3390/s21020361 (PMC7825773; doi:10.3390/s21020361)

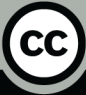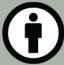

BY

Supplement: Supplementary file 1 [file sensors-21-00361-s001.zip › Definitions/logo-ccby-eps-converted-to.pdf]

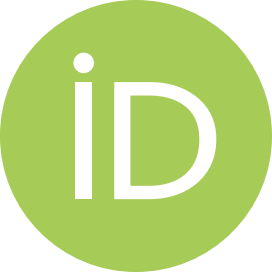

Supplement: Supplementary file 1 [file sensors-21-00361-s001.zip › Definitions/logo-orcid-eps-converted-to.pdf]

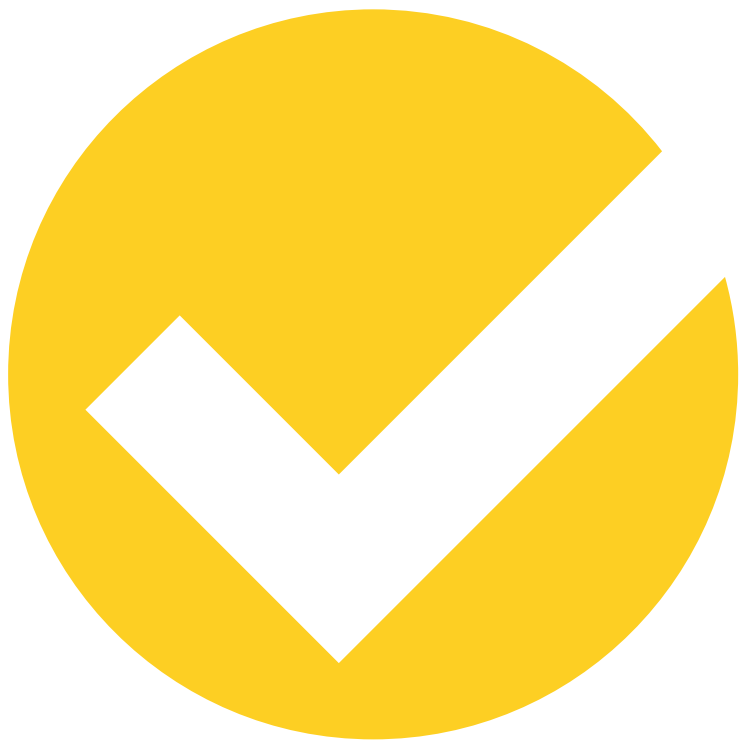

check for  
updates

Supplement: Supplementary file 1 [file sensors-21-00361-s001.zip › Definitions/logo-updates.pdf]

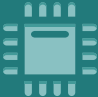

*sensors*

Supplement: Supplementary file 1 [file sensors-21-00361-s001.zip › Definitions/sensors-logo-eps-converted-to.pdf]

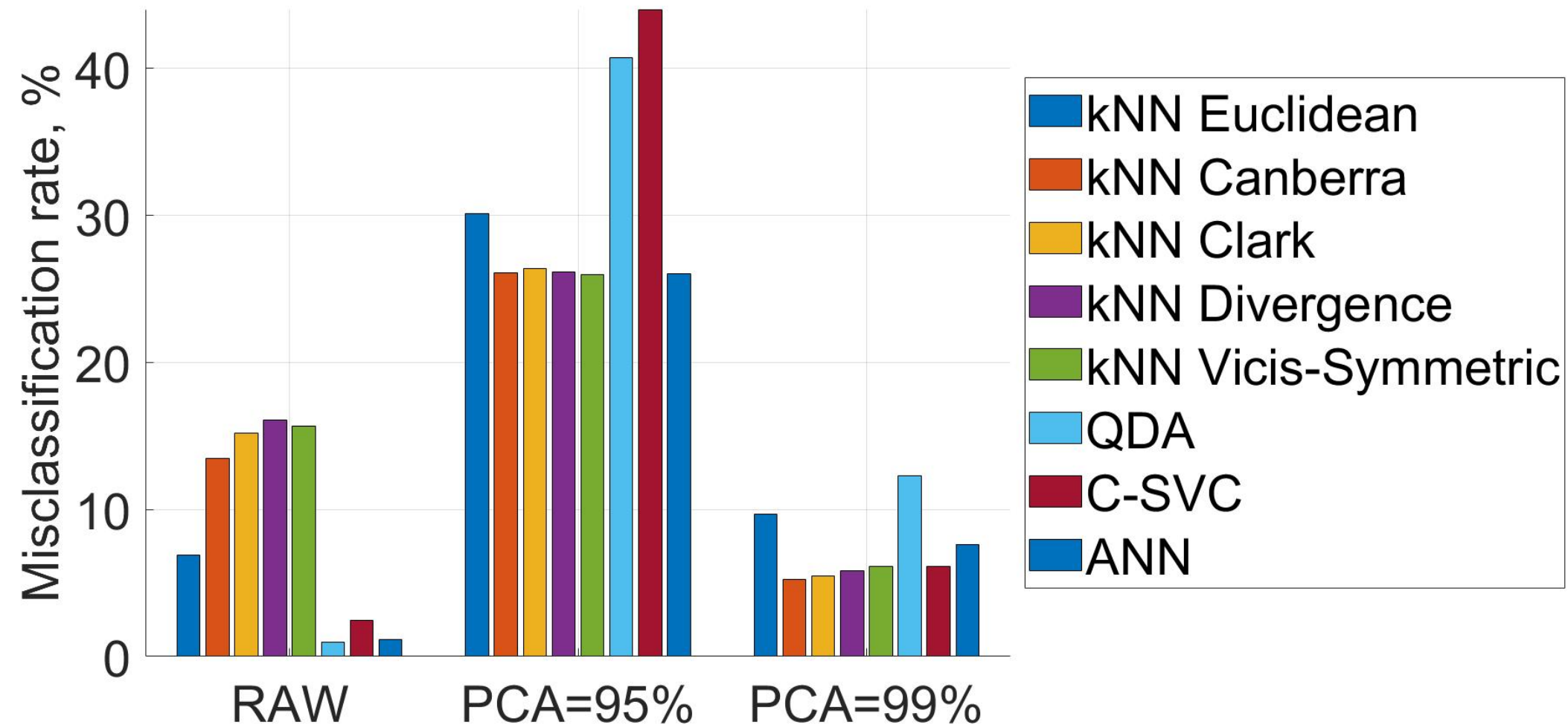

Supplement: Supplementary file 1 [file sensors-21-00361-s001.zip › fig/AverageErrors.pdf]

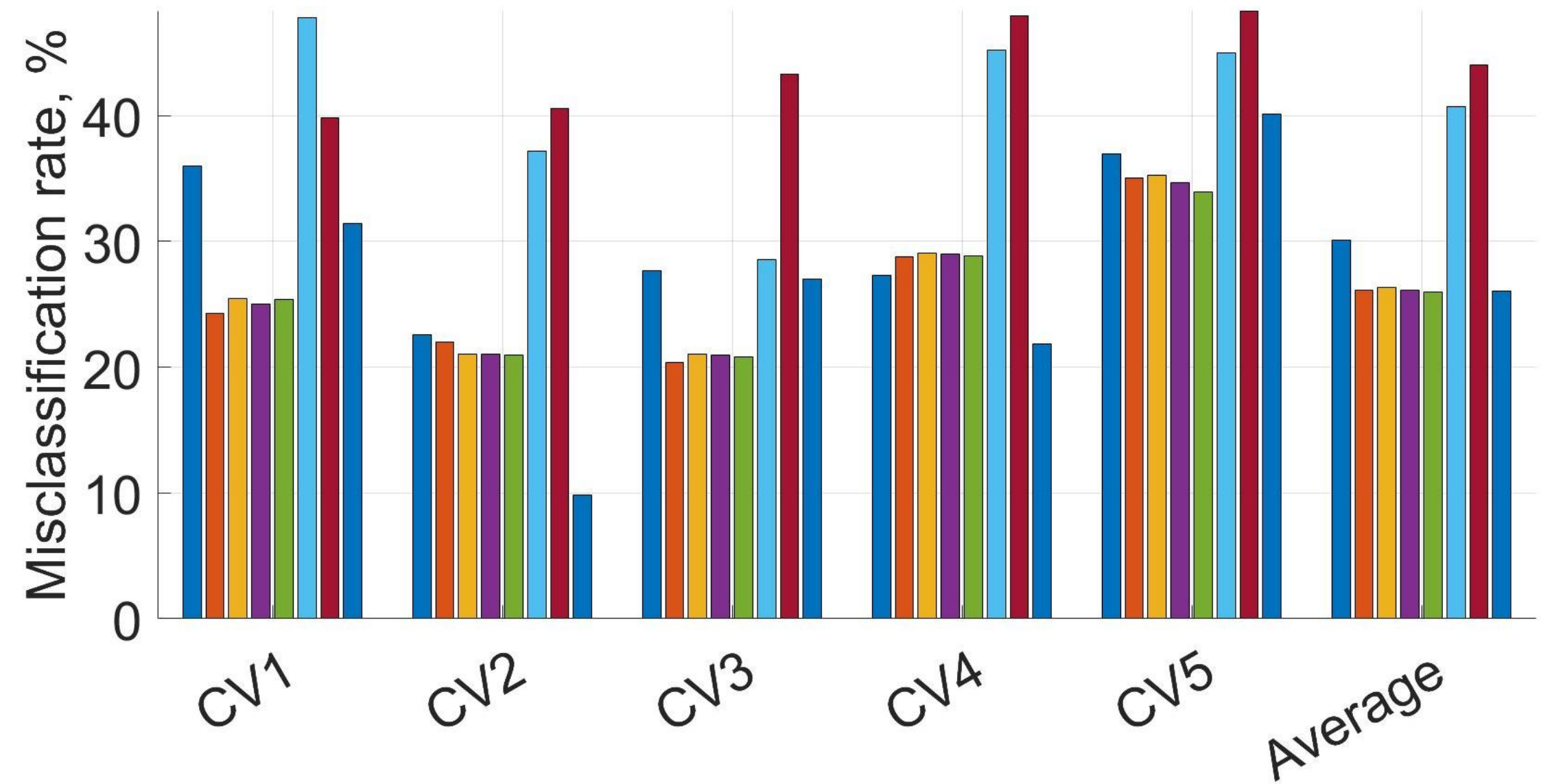

Supplement: Supplementary file 1 [file sensors-21-00361-s001.zip › fig/CVPlotsPCA95noLegend.pdf]

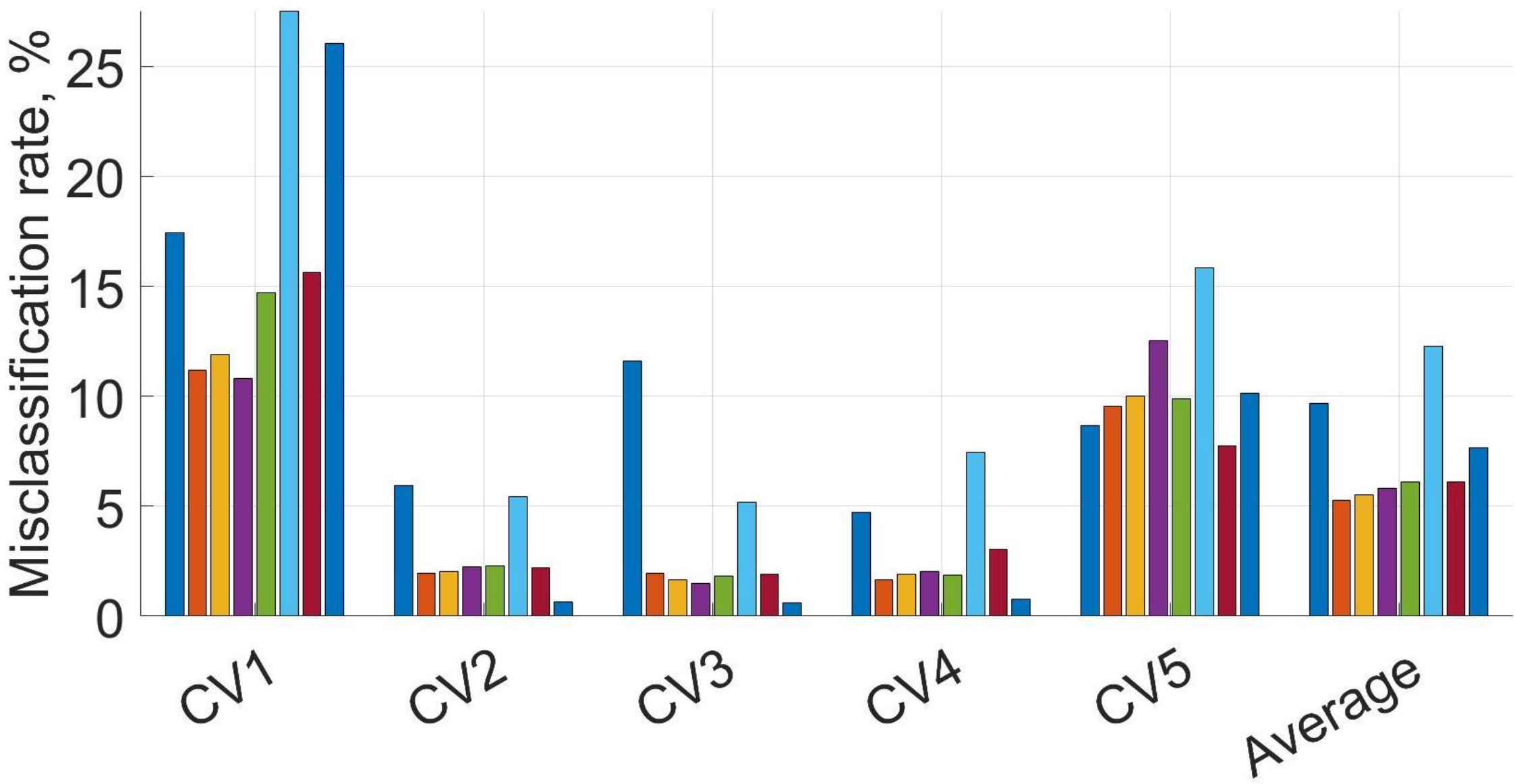

Supplement: Supplementary file 1 [file sensors-21-00361-s001.zip › fig/CVPlotsPCA99noLegend.pdf]

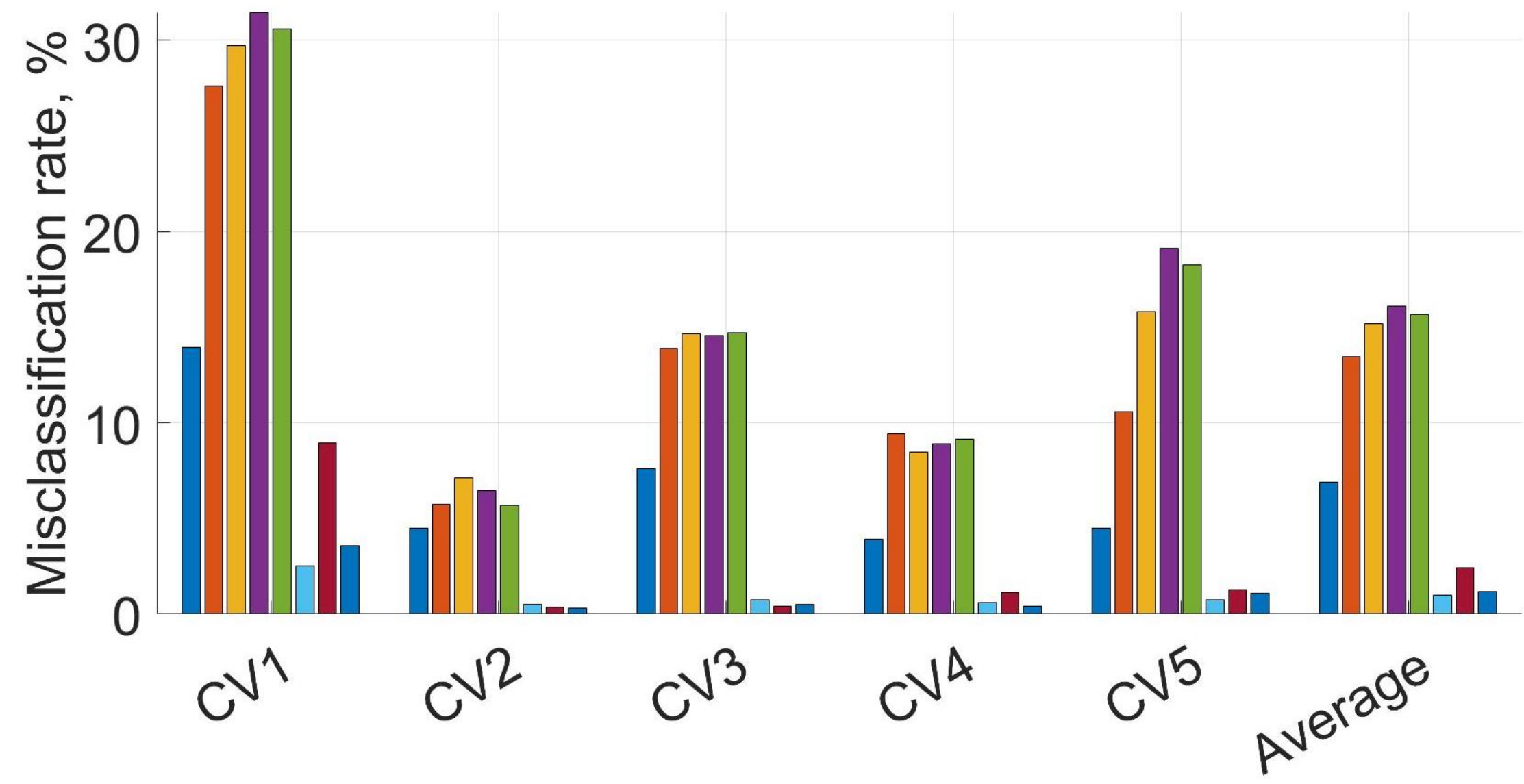

Supplement: Supplementary file 1 [file sensors-21-00361-s001.zip › fig/CVPlotsRAWnoLegend.pdf]

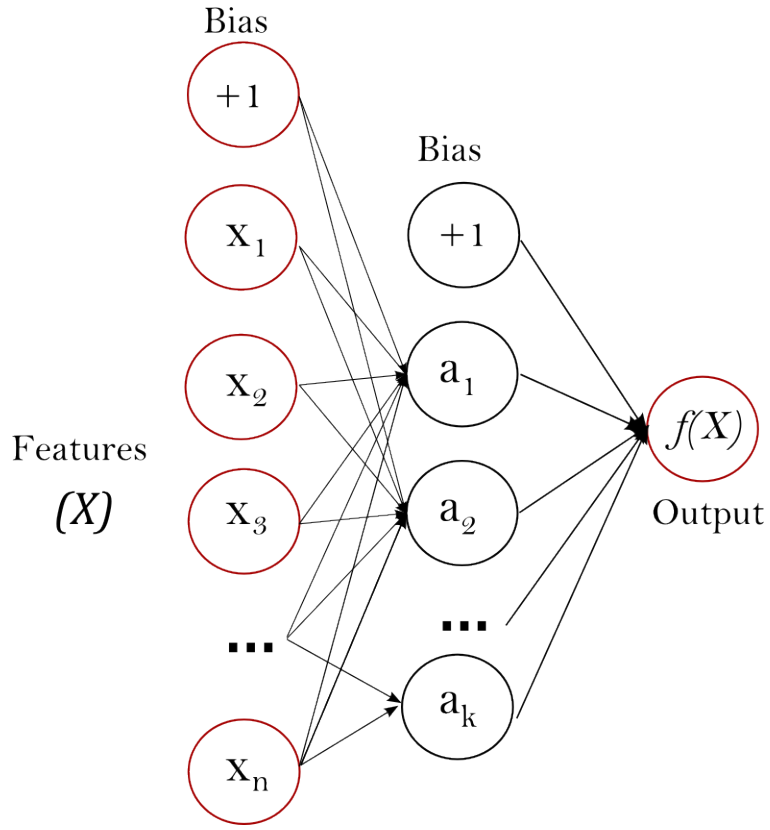

Supplement: Supplementary file 1 [file sensors-21-00361-s001.zip › fig/MLPClassifier.pdf]

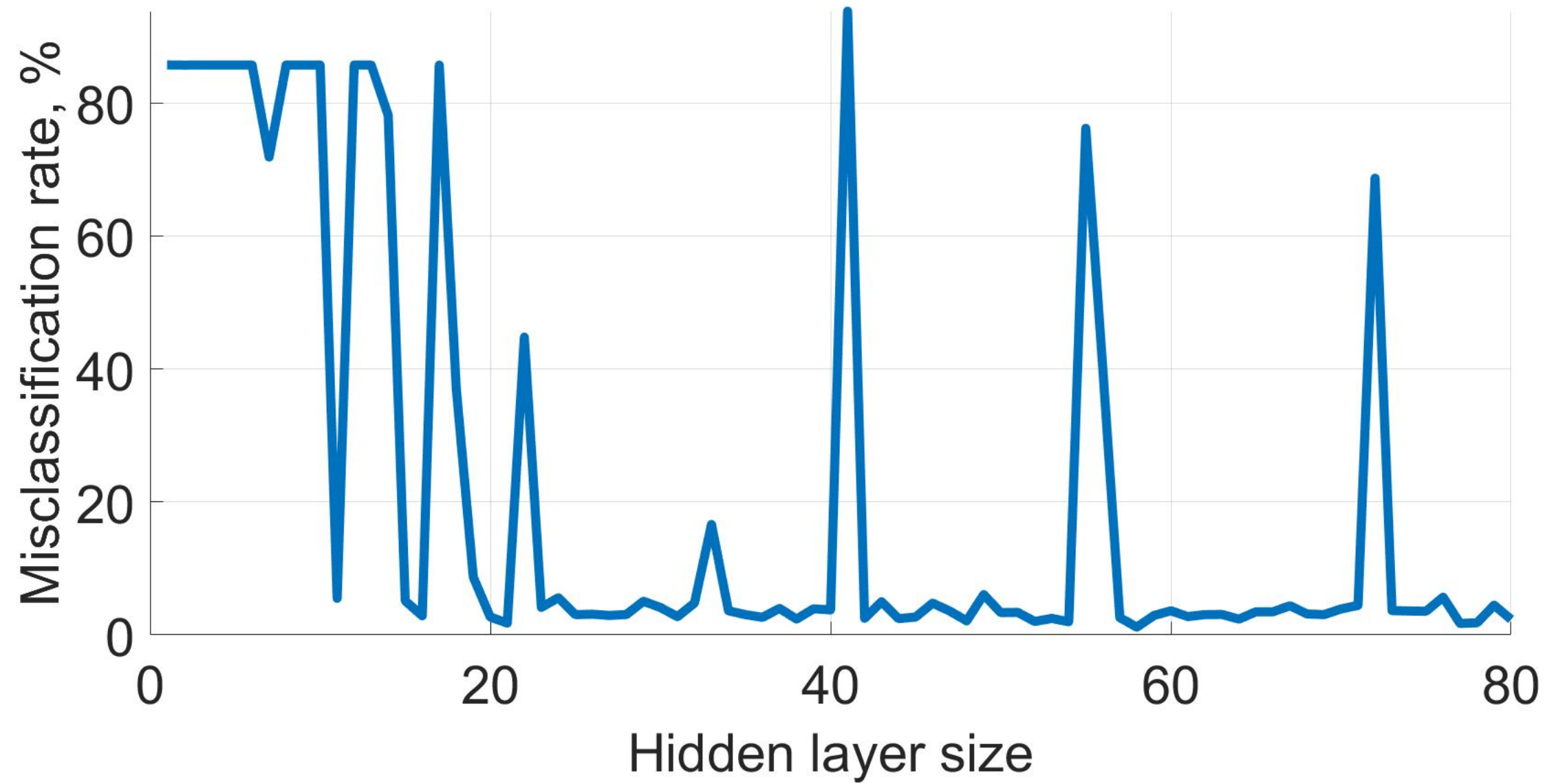

Supplement: Supplementary file 1 [file sensors-21-00361-s001.zip › fig/NNErrors.pdf]

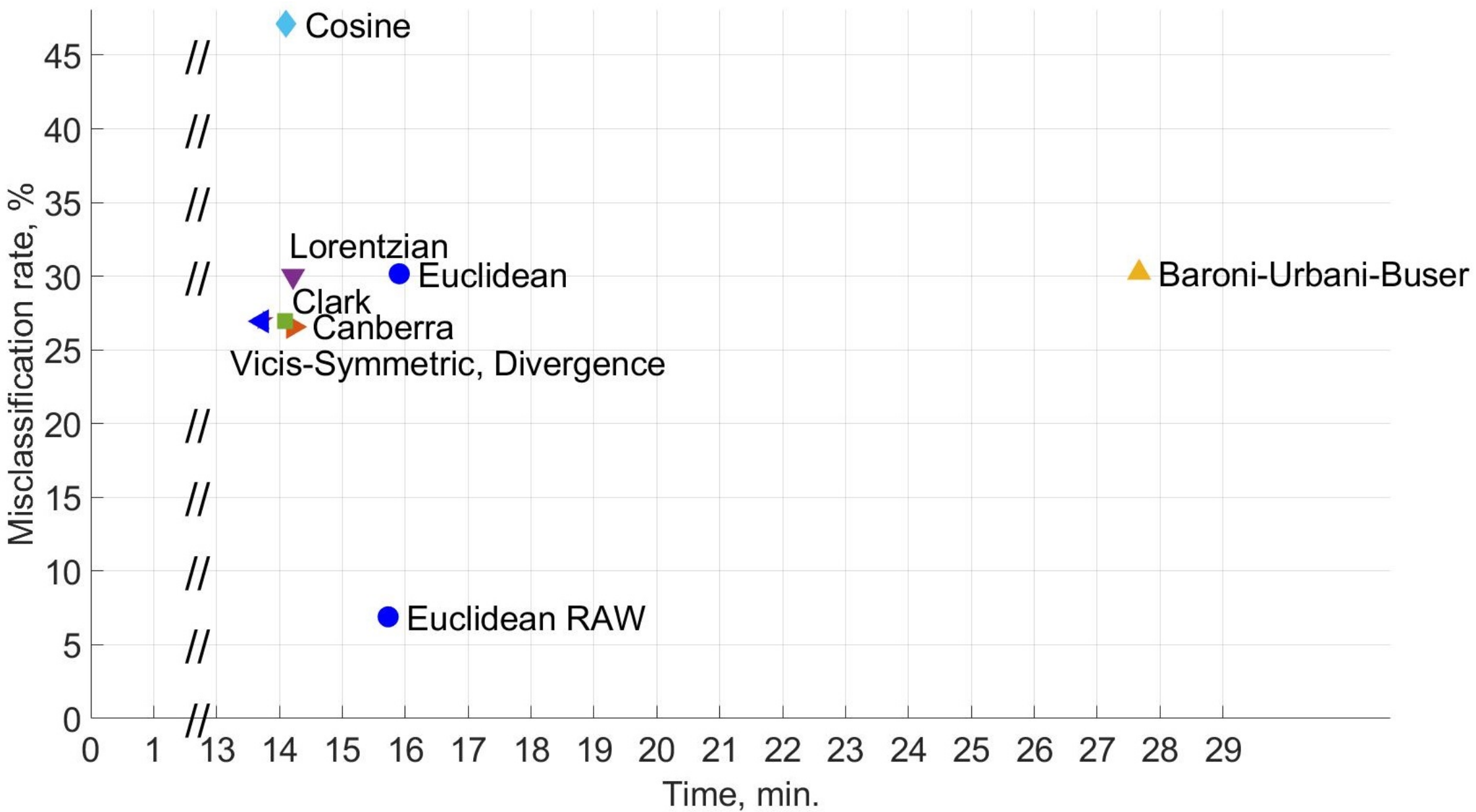

Supplement: Supplementary file 1 [file sensors-21-00361-s001.zip › fig/ParetoNormPCA94.pdf]

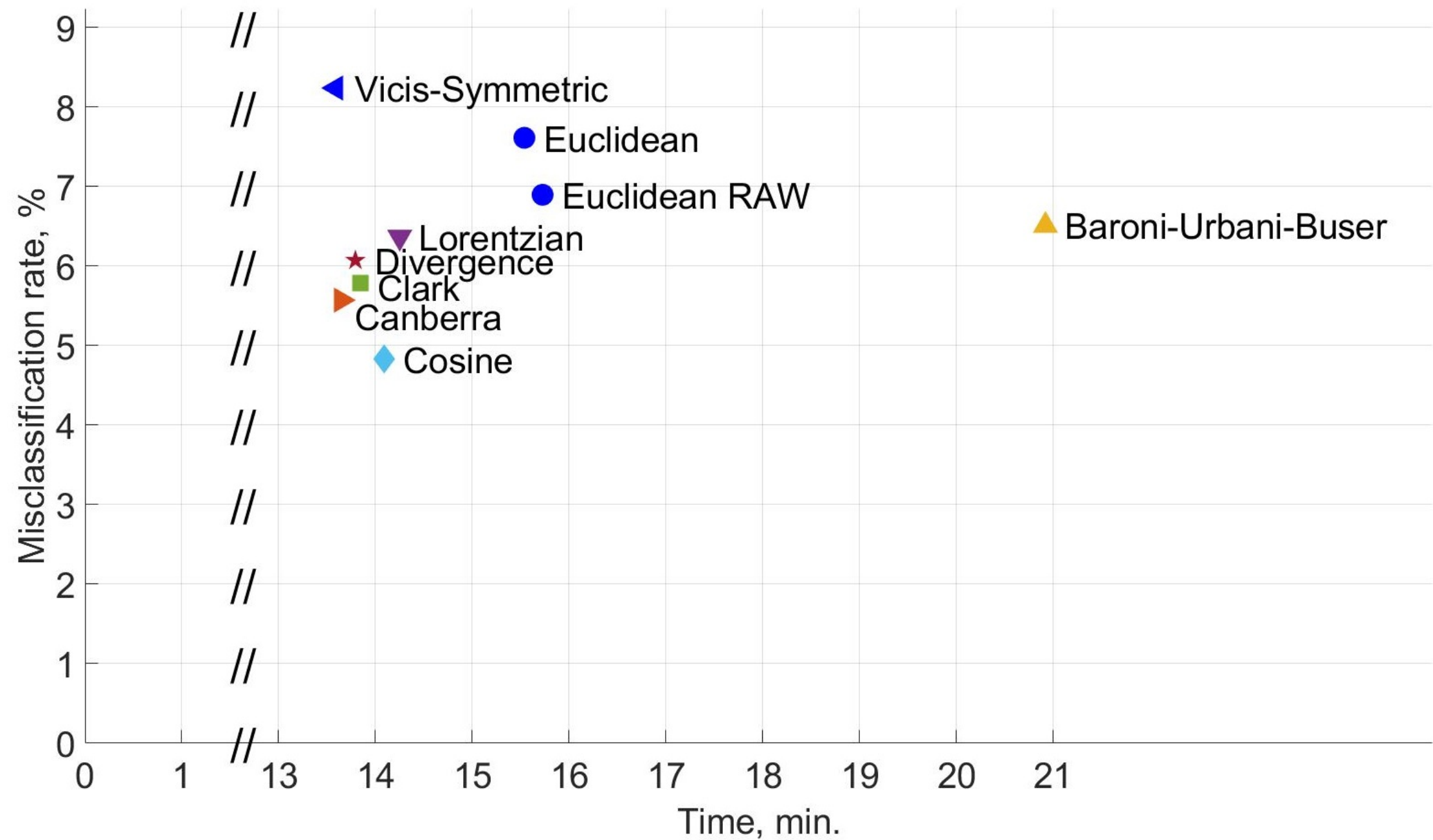

Supplement: Supplementary file 1 [file sensors-21-00361-s001.zip › fig/ParetoNormPCA99.pdf]

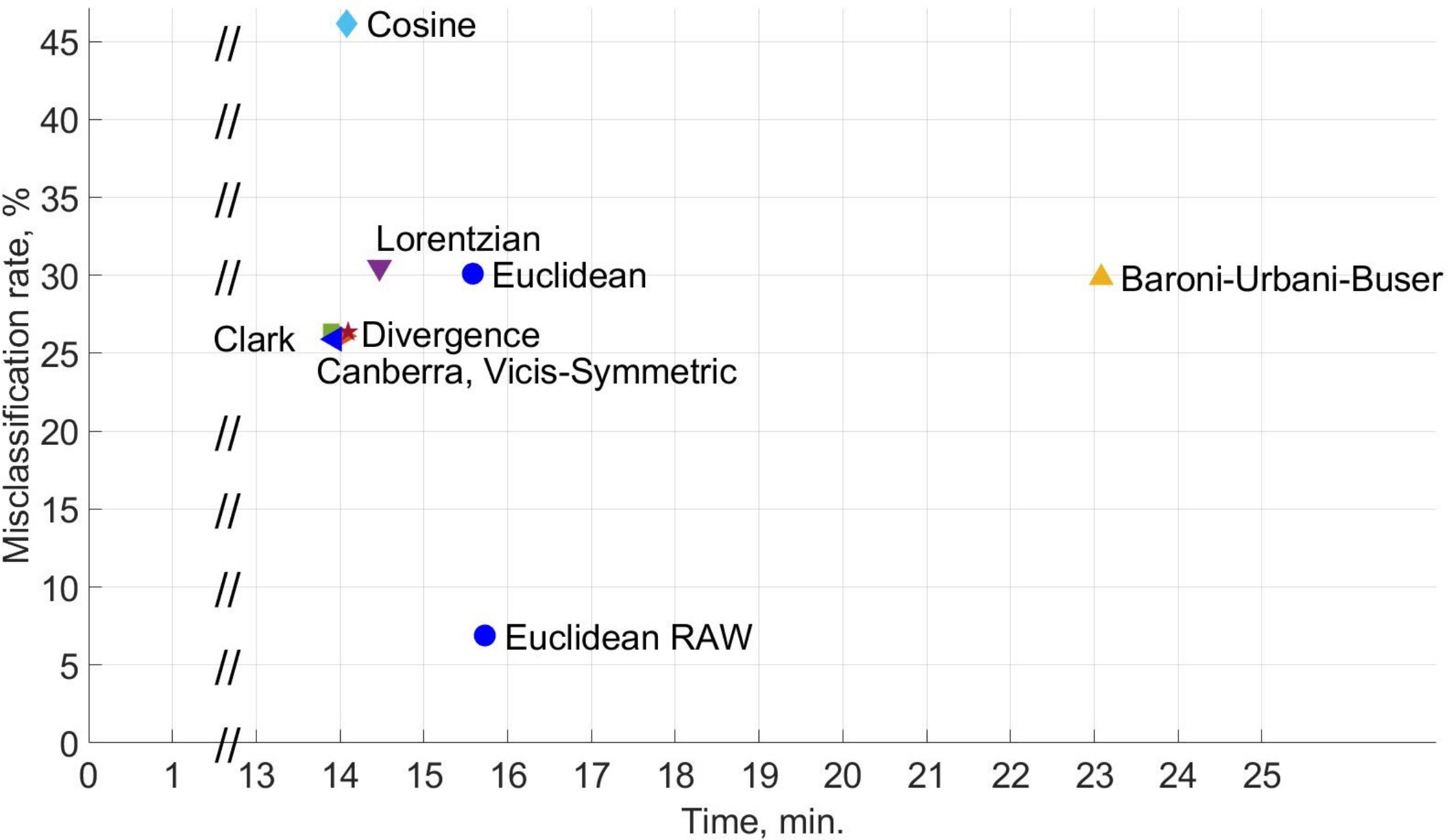

Supplement: Supplementary file 1 [file sensors-21-00361-s001.zip › fig/ParetoPCA94.pdf]

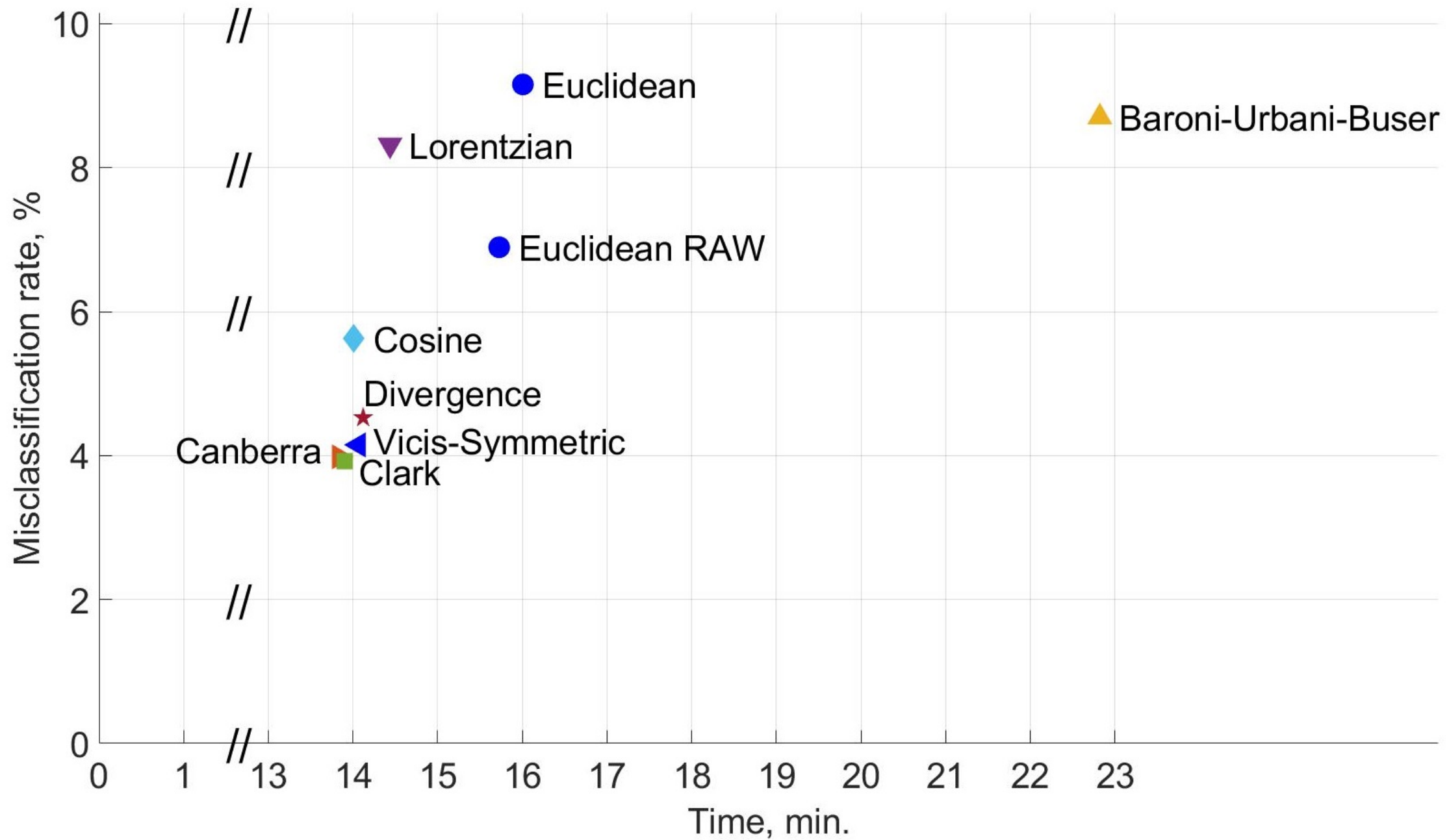

Supplement: Supplementary file 1 [file sensors-21-00361-s001.zip › fig/ParetoPCA99.pdf]

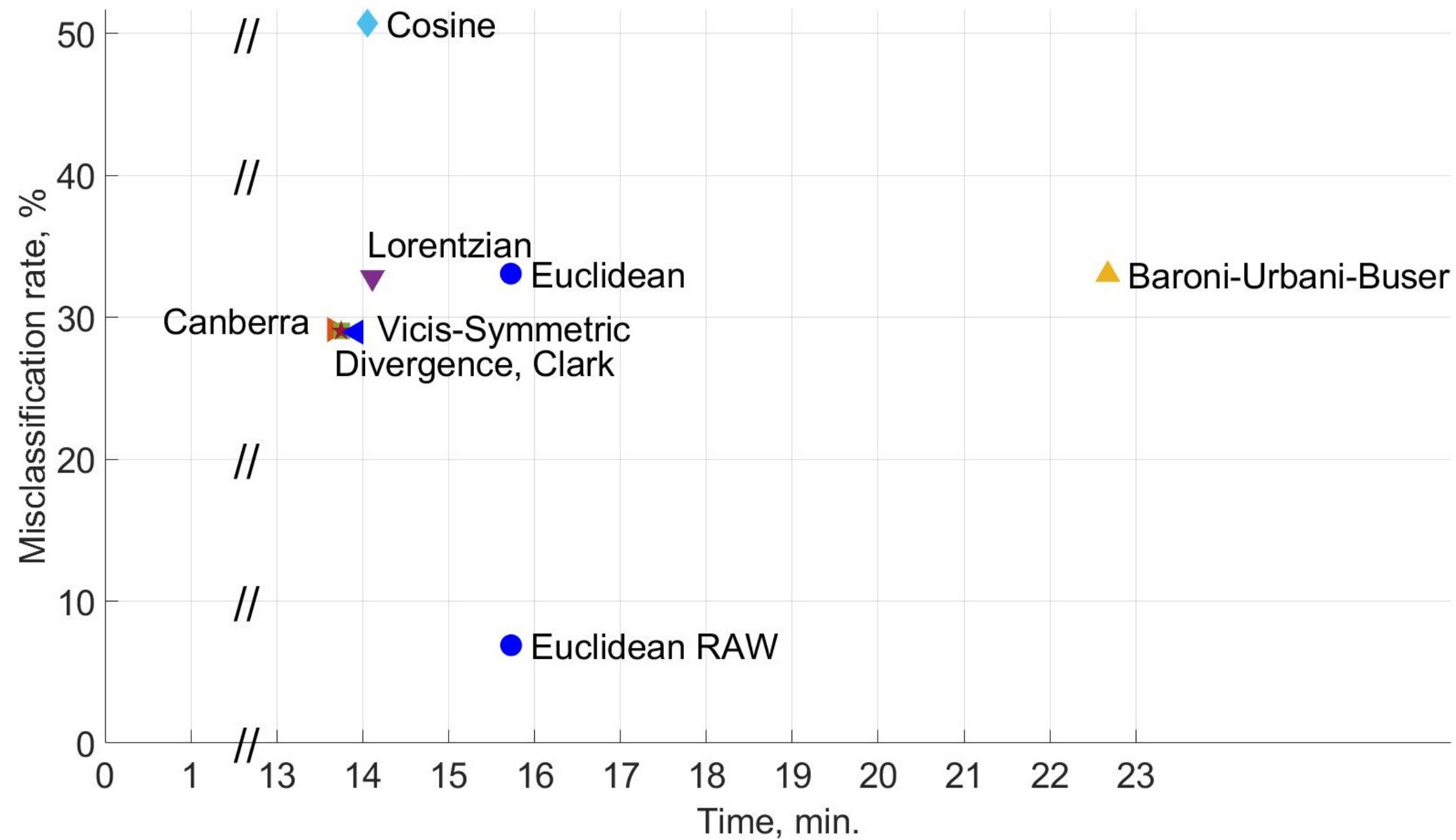

Supplement: Supplementary file 1 [file sensors-21-00361-s001.zip › fig/ParetoSmooth11NormPCA94.pdf]

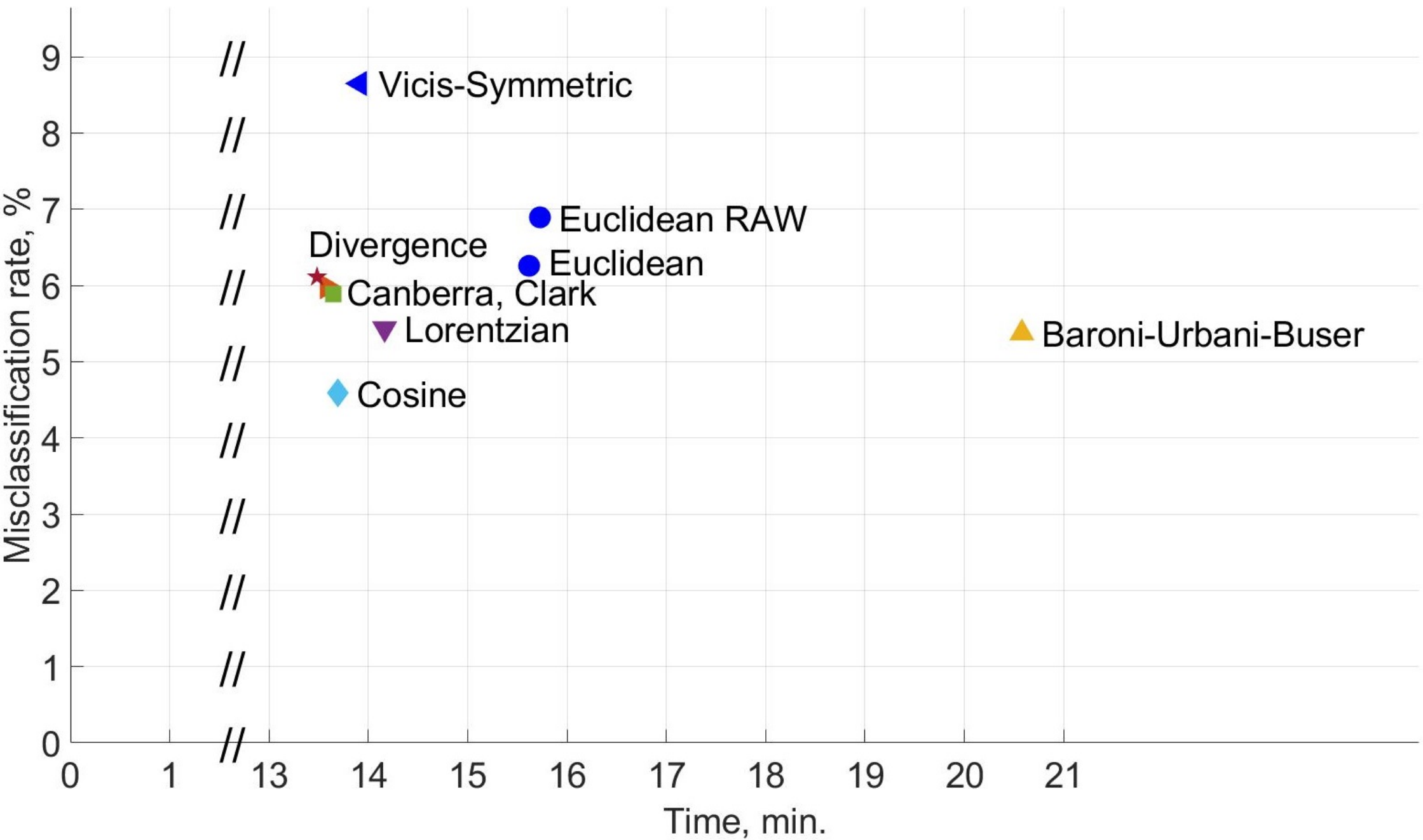

Supplement: Supplementary file 1 [file sensors-21-00361-s001.zip › fig/ParetoSmooth11NormPCA99.pdf]

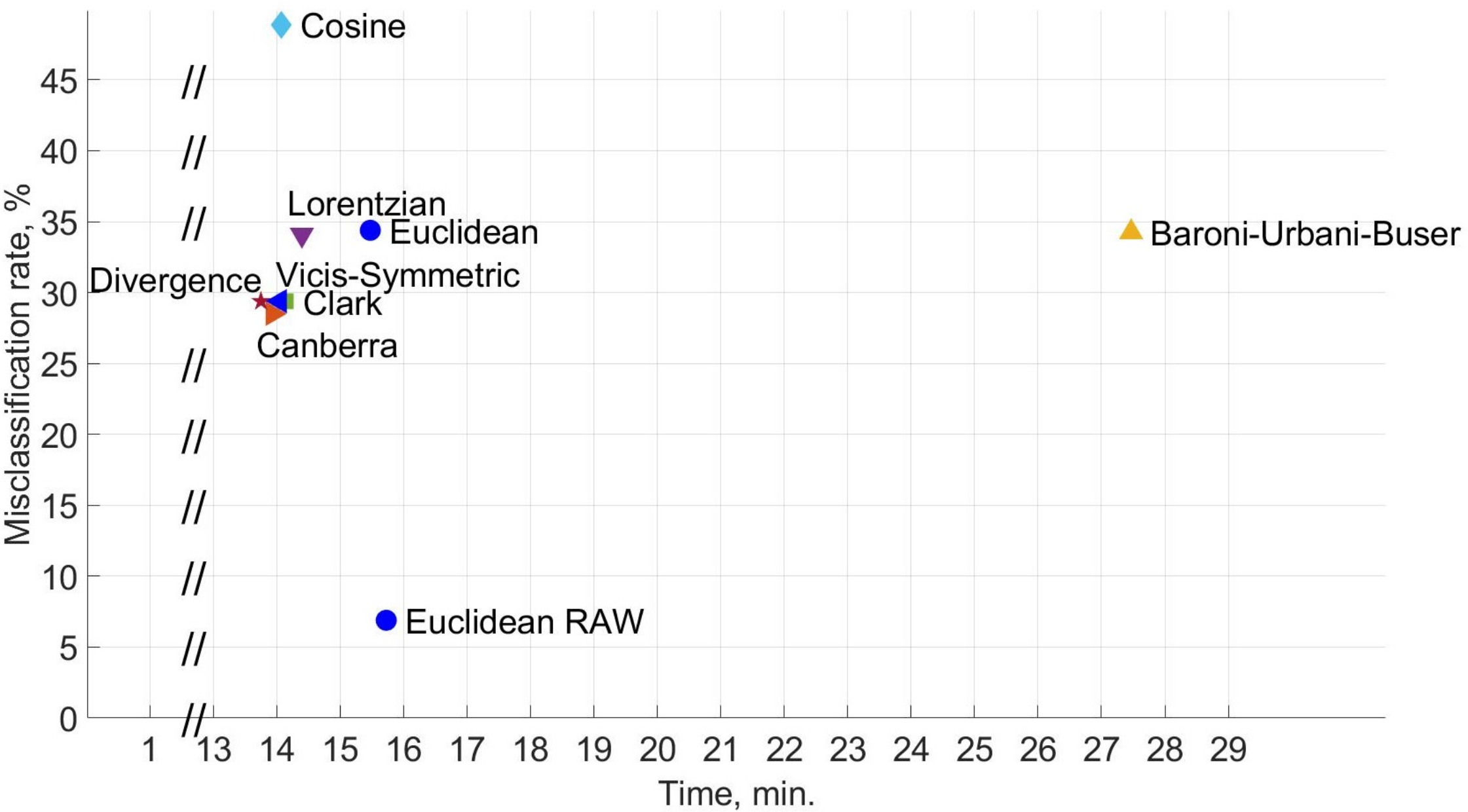

Supplement: Supplementary file 1 [file sensors-21-00361-s001.zip › fig/ParetoSmooth5NormPCA94.pdf]

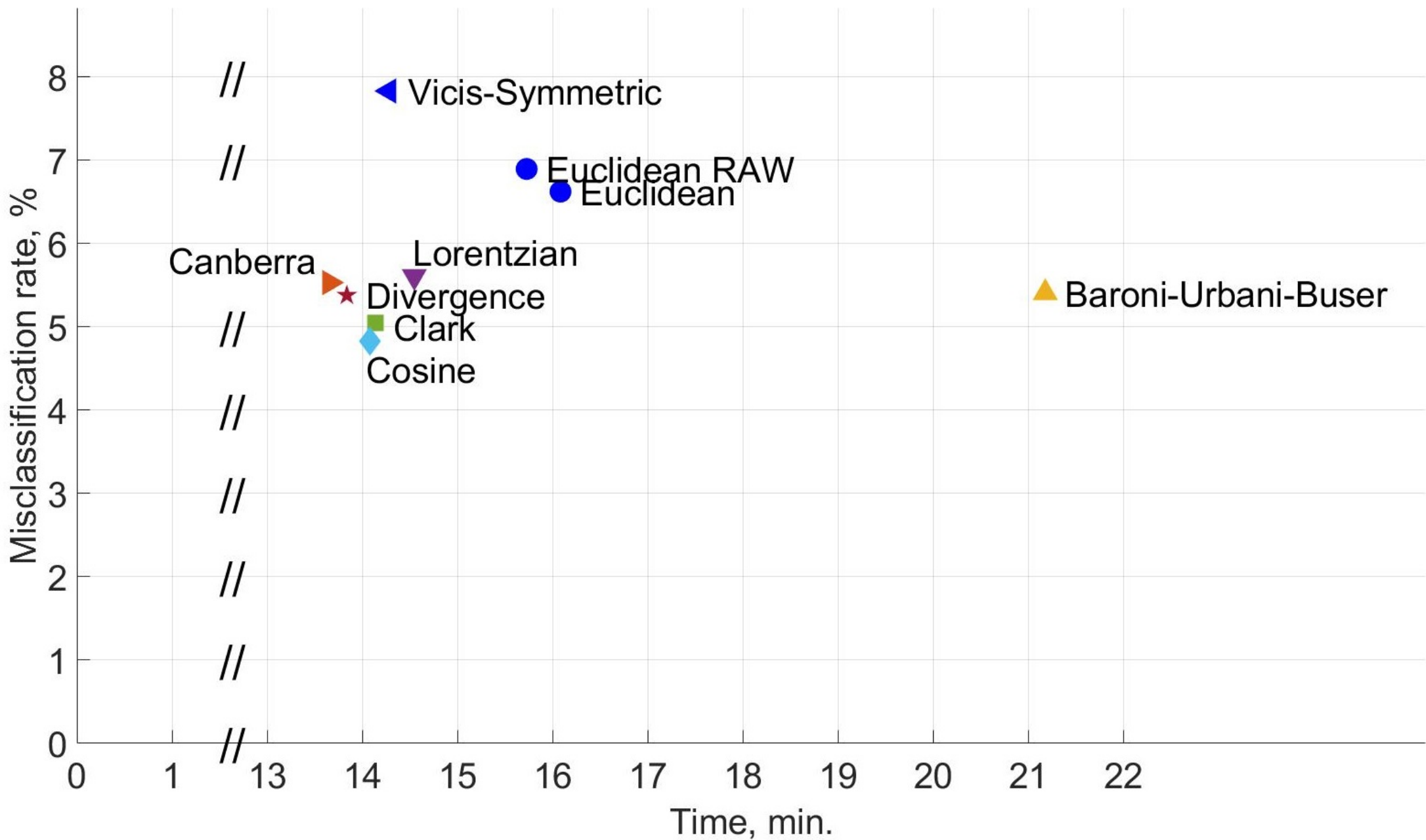

Supplement: Supplementary file 1 [file sensors-21-00361-s001.zip › fig/ParetoSmooth5NormPCA99.pdf]

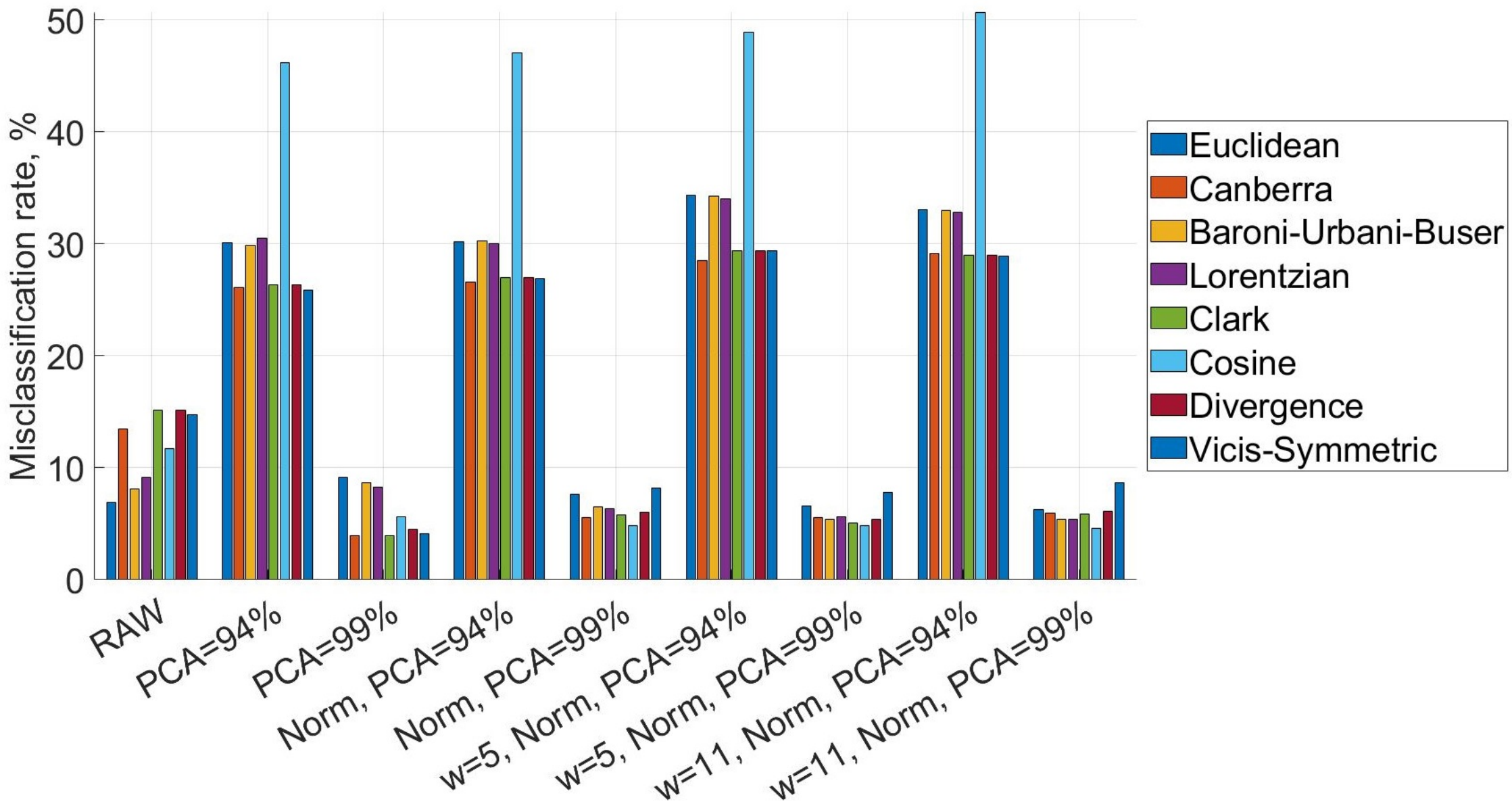

Supplement: Supplementary file 1 [file sensors-21-00361-s001.zip › fig/RawPCAtest.pdf]

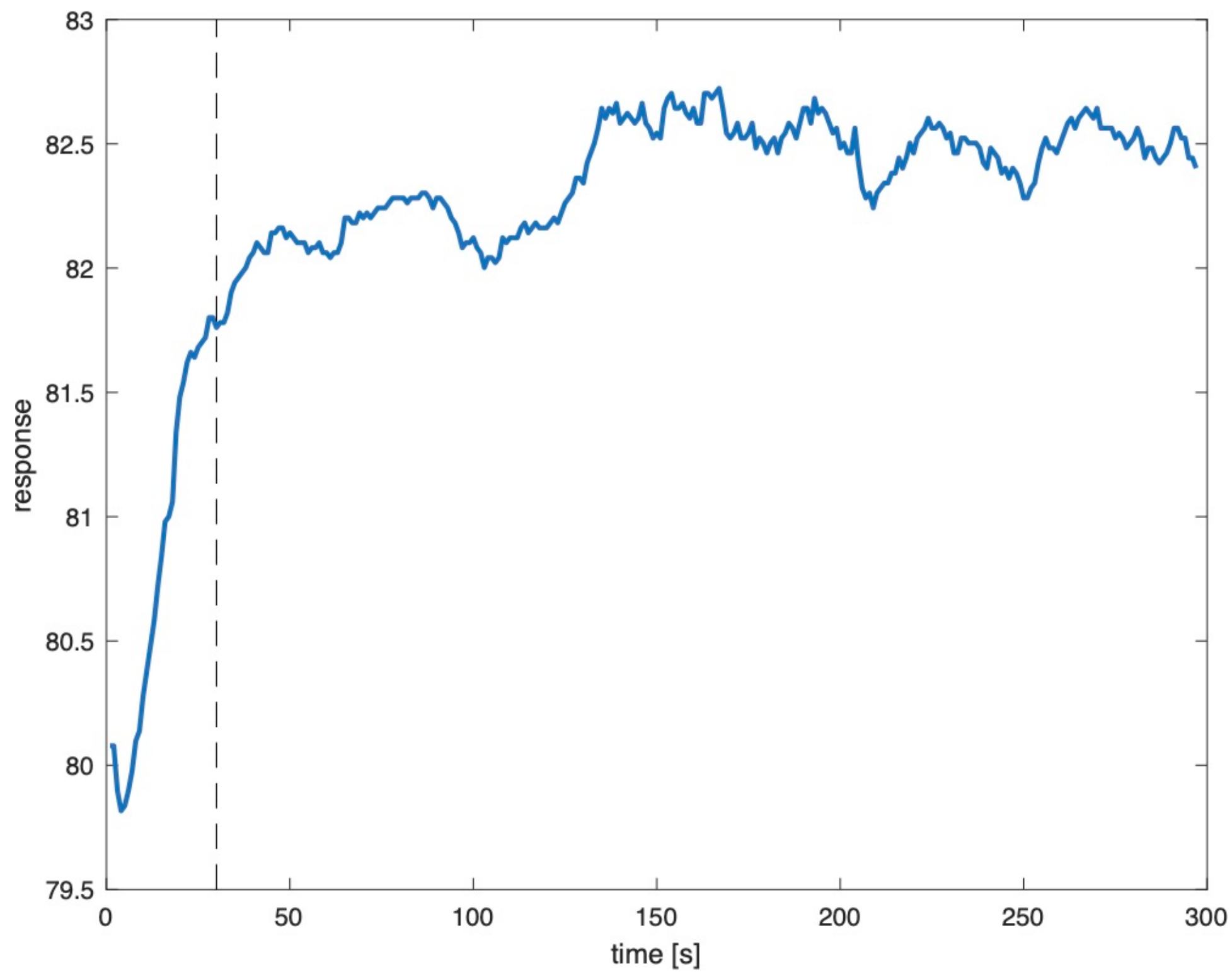

Supplement: Supplementary file 1 [file sensors-21-00361-s001.zip › fig/transientphase.pdf]
